# VPS35 regulates parkin substrate AIMP2 toxicity by facilitating lysosomal clearance of AIMP2

**DOI:** 10.1038/cddis.2017.157

**Published:** 2017-04-06

**Authors:** Seung Pil Yun, Hyojung Kim, Sangwoo Ham, Seung-Hwan Kwon, Gum Hwa Lee, Joo-Ho Shin, Sang Hun Lee, Han Seok Ko, Yunjong Lee

**Affiliations:** 1 Neuroregeneration and Stem Cell Programs, Institute for Cell Engineering, The Johns Hopkins University School of Medicine, Baltimore, MD, USA; 2 Department of Neurology, The Johns Hopkins University School of Medicine, Baltimore, MD, USA; 3 Adrienne Helis Malvin Medical Research Foundation, New Orleans, LA, USA; 4 Division of Pharmacology, Department of Molecular Cell Biology, Sungkyunkwan University School of Medicine, Samsung Biomedical Research Institute, Suwon, South Korea; 5 College of Pharmacy, Chosun University, Gwangju, Republic of Korea; 6 Medical Science Research Institute, Soonchunhyang University, Seoul Hospital, Seoul, Republic of Korea; 7 Diana Helis Henry Medical Research Foundation, New Orleans, LA, USA

## Abstract

Vacuolar protein sorting-associated protein 35 (VPS35) is involved in retrograde transport of proteins from endosomes to trans-Golgi network. Gene mutations in VPS35 are linked to autosomal dominant late-onset Parkinson's disease (PD). Although the identification of VPS35 mutations has provided novel insight about its interactions with several PD-associated genes including leucine-rich repeat kinase 2 (LRRK2) and *α*-synuclein, little information is available about the molecular mechanisms of cell death downstream of VPS35 dysfunction. In this study, we showed that VPS35 has a role in the lysosomal degradation of parkin substrate aminoacyl tRNA synthetase complex-interacting multifunctional protein 2 (AIMP2), of which accumulation leads to poly(ADP-ribose) polymerase-1 (PARP1)-dependent cell death. VPS35 was co-immunoprecipitated with AIMP2, as well as lysosome-associated membrane protein-2a (Lamp2a). Interestingly, this association was disrupted by PD-associated VPS35 mutant D620N. VPS35 overexpression prevented AIMP2-potentiated cell death and PARP1 activation in SH-SY5Y cells. More importantly, knockdown of VPS35 led to PARP1 activation and cell death, which was AIMP2 dependent. These findings provide new mechanistic insights into the role of VPS35 in the regulation of AIMP2 levels and cell death. As AIMP2 accumulation was reported in PD patient's brains and involved in dopaminergic cell death, identification of VPS35 as a novel regulator of AIMP2 clearance via lysosomal pathway provides alternative venue to control dopaminergic cell death in PD.

Degeneration of midbrain dopaminergic neurons contributes to cardinal motor deficits in Parkinson's disease (PD). PD-linked mutations in several genes (parkin, *α*-synuclein, PINK1, DJ-1, LRRK2 and vacuolar protein sorting-35 (VPS35)) have been reported, providing clues to the molecular mechanisms involved in dopaminergic neuronal dysfunction or demise in PD patients.^1, 2, 3, 4, 5^ Parkin is an E3 ubiquitin ligase whose mutations or phosphorylation by stress-activated kinase c-Abl inhibits its ligase activity and neuroprotective function in dopaminergic neurons.^2^ Accumulation of parkin substrates is responsible for PD-associated pathological changes downstream of parkin inactivation.^6, 7, 8^ For example, parkin-interacting substrate (PARIS) can lead to peroxisome proliferator-activated receptor gamma coactivator 1-alpha repression and defects in antioxidant defense in PD animal models.^6^ Accumulation of Pael receptor, another parkin substrate, can increase endoplasmic reticulum (ER) stress, which is associated with pathological changes in PD.^8^ Recently, more direct link of PD to dopaminergic cell death downstream of parkin inactivation has been reported. Aminoacyl tRNA synthetase complex-interacting multifunctional protein 2 (AIMP2) is a pathogenic parkin substrate that accumulates in animal models of parkin inactivation and postmortem PD patient brains.^9, 10^ Conditional transgenic mouse expressing AIMP2 in brains has revealed that AIMP2 accumulation alone is sufficient for age-dependent and -selective dopaminergic neurodegeneration.^10^ Importantly, nuclear translocation of AIMP2 and interaction with the nuclear enzyme poly(ADP-ribose) polymerase-1 (PARP1) lead to overproduction of poly(ADP-ribose) (PAR) that is responsible for PAR-dependent cell death (parthanatos).^10^ As PAR-dependent cell death because of AIMP2 accumulation seems to be involved in dopaminergic loss in PD, it would be important to understand the molecular mechanisms underlying AIMP2 clearance to prevent progressive neurodegeneration in PD.

As a lysine 48 (K48) ubiquitin substrate of parkin, AIMP2 is polyubiquitinated and targeted to the proteasome for degradation.^9, 10, 11, 12^ Thus, MG132 inhibition of proteasome results in the elevation of AIMP2 protein levels. Interestingly, previous study has also shown that both lysosomal inhibition and proteasome inhibition have hampered AIMP2 degradation to some extent,^12^ suggesting that AIMP2, in addition to parkin-mediated proteasomal degradation, could be regulated through lysosomal degradation by yet unknown molecular pathway.

Autosomal dominant mutations in VPS35 have been recently identified by exome sequencing. They are responsible for late-onset PD in multi-incidence families.^13, 14^ Clinical manifestation in PD patients with VPS35 mutations is quite similar to sporadic PD cases with late-onset motor symptoms, including Lewy body pathology and good L-DOPA responsiveness.^14^ VPS35 is a component of retromer complex that functions in endosome-to-golgi retrieval of membrane proteins, endosomal recycle and others.^15, 16, 17^ Interestingly, VPS35 downregulation is clinically associated with neurodegenerative diseases including PD and Alzheimer's disease (AD).^18, 19^ Consistent with this notion, VPS35 ablation in dopaminergic neurons results in PD-associated deficits in mice, such as *α*-synuclein accumulation, loss of dopamine neurons and motor impairment.^20, 21^ Absence of VPS35 renders yeast more vulnerable to protein aggregation-induced toxicity by eukaryotic translation initiation factor 4 gamma 1 (EIF4G1) overexpression.^22^ Conversely, overexpression of WT VPS35 rescues locomotor deficits and reduction of lifespan in leucine-rich repeat kinase 2 (LRRK2) transgenic PD fly models.^23^ VPS35 also protects neurons against mitochondrial toxin 1-methyl-4-phenylpyridinium (MPP^+^)-induced oxidative stress.^24^ Dysfunction of VPS35 by reduction of its expression levels or PD-linked mutations results in abnormal recycling or subcellular targeting of its substrates.^17, 20, 25^ For instance, VPS35 deficits lead to abnormal delivery of lysosome proteases because of defective targeting of cation-independent mannose 6-phosphate receptor, a cargo of VPS35/retromer.^17, 25^ It has also been shown that impaired endosome-to-golgi retrieval of lysosome-associated membrane glycoprotein-2a (Lamp2a) is responsible for lysosomal degradation of available Lamp2a pool in dopamine neurons of VPS35 deletion.^20^ As Lamp2a mediates *α*-synuclein degradation via chaperone-mediated autophagy (CMA), downregulation of lamp2a following VPS35 deficits can lead to accumulation of *α*-synuclein.^20^

Here, we identified VPS35 as a novel regulator of AIMP2 by lysosomal clearance. VPS35 overexpression reduced endogenous AIMP2 levels and prevented AIMP2 accumulation. VPS35 interacted with AIMP2 via Lamp2a, suggesting CMA-mediated degradation of AIMP2. VPS35 knockdown resulted in AIMP2 accumulation and sensitized cells to parthanatic cell death. Our data indicated that accumulation of AIMP2 in VPS35 dysfunction could contribute to dopaminergic cell toxicity in PD. Our finding also provided a novel pathway of AIMP2 degradation that could be used to control AIMP2 accumulation and dopaminergic cell death in PD.

## Results

### VPS35 facilitates AIMP2 degradation via lysosome

**Figure 1:**
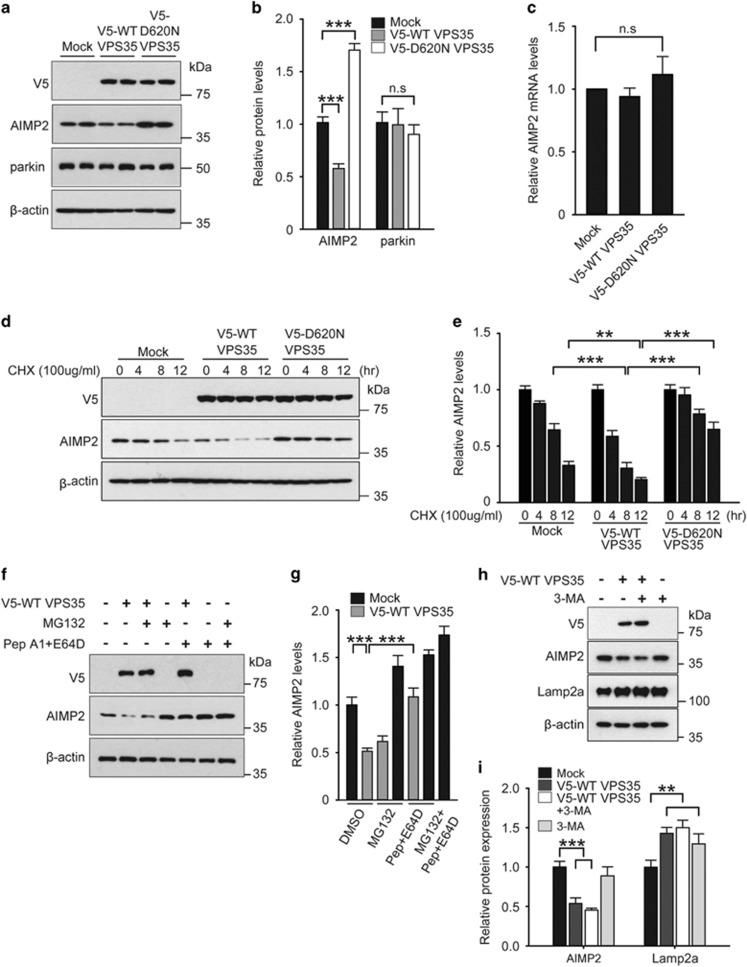
VPS35 regulates AIMP2 clearance via lysosomal degradation. (**a**) SH-SY5Y cells were transfected with mock, V5-WT VPS35, or V5-D620N VPS35 plasmids. At 48 h post transfection, total protein was extracted and immunoblotted with V5, AIMP2 and parkin antibody to monitor their levels. *β*-Actin was used as an internal loading control. (**b**) Normalized quantification of AIMP2 and parkin levels in the indicated samples are expressed as a bar graph (*n*=4 per group). (**c**) Quantification of relative *AIMP2* mRNA levels in SH-SY5Y cells transfected with mock, WT VPS35 or D620 VPS35 and determined by real-time quantitative PCR. *GAPDH* mRNA level was used as internal loading control (*n*=5 per group). (**d**) Western blots of steady-state levels of AIMP2 at the indicated time after cycloheximide (CHX) treatment. SH-SY5Y cells were transfected with mock V5-WT VPS35 or V5-D620N VPS35. *β*-Actin was used as an internal loading control. (**e**) Normalized levels of AIMP2 in SH-SY5Y cells transfected with indicated constructs (*n*=4). (**f**) SH-SY5Y cells were transient transfected with mock or V5-WT VPS35. At 48 h post transfection, MG132 (a proteasome inhibitor, 20 *μ*M, 8 h) and/or pepstatin A1 and E64D (lysosome inhibitors, 10 *μ*g/ml, 4 h) were used to treat cells. Total protein was extracted and immunoblotted with V5 and AIMP2 antibody to determine their levels. *β*-Actin was used as an internal loading control. (**g**) Normalized levels of AIMP2 in SH-SY5Y cells transfected with mock or V5-VPS35 constructs followed by treatment with proteasome or lysosome inhibitors (*n*=4 per group). (**h**) SH-SY5Y cells were transiently transfected with mock or V5-VPS35. At 48 h post transfection, cells were treated with 3-MA (10 mM, 8 h). Total protein was extracted and immunoblotted with V5, AIMP2 and Lamp2a antibody to monitor their levels. *β*-Actin was used as an internal loading control. (**i**) Normalized levels of AIMP2 and Lamp2a in SH-SY5Y cells transfected with mock or V5-VPS35 constructs followed by treatment with macroautophagy inhibitor, 3-MA (*n*=3 per group). Two-way ANOVA followed by Bonferroni post-hoc analysis was used multiple group comparison. **P<0.01, ***P<0.001 *versus* control or WT VPS35 alone. n.s, not significant

AIMP2 accumulation is strongly associated with dopaminergic cell death in PD.^9, 10, 11, 12^ To identify the potential role of VPS35 in regulating pathogenic parkin substrate AIMP2, we performed a small-scale screening in SH-SY5Y cells transiently transfected with V5-WT VSP35 or the PD-linked mutant version V5-D620N VPS35. Overexpression of WT VPS35 reduced endogenous AIMP2 levels (Figures 1a and b). However, overexpression of D620N VPS35 increased the endogenous levels of AIMP2 (Figures 1a and b). Alteration of AIMP2 expression by VPS35 was not mediated by the E3 ubiquitin ligase parkin because VPS35 did not affect endogenous parkin expression levels (Figures 1a and b). Moreover, AIMP2 mRNA level did not change by the overexpression of either WT or D620N VPS35 (Figure 1c), indicating that VPS35 regulates AIMP2 via mechanisms downstream of transcription. To further determine the degradation rate of endogenous AIMP2 by VPS35 overexpression, endogenous AIMP2 levels were monitored over time after treatment with cycloheximide, an inhibitor of protein biosynthesis. Overexpression of WT VPS35 accelerated the degradation of AIMP2. However, AIMP2 clearance was inhibited by overexpression of D620N VPS35 (Figures 1d and e).

Next, we sought to determine how VPS35 facilitated AIMP2 clearance by using pharmacological inhibitors of either proteasome or lysosome. Consistent with previous reports, both proteasome inhibitor MG132 and lysosome inhibitors Pepstatin A1 and E64D resulted in accumulation of steady-state levels of endogenous AIMP2 (Figures 1f and g). These results indicate that AIMP2 clearance is mediated by both proteasome and lysosome degradation pathways. Interestingly, treatment with lysosomal inhibitor Pepstatin A1 and E64D completely blocked the accelerated degradation of AIMP2 by VPS35 overexpression, whereas MG132 treatment failed to reverse the effect of VPS35 on AIMP2 degradation (Figures 1f and g). Consistent with the notion that VPS35 influences lysosomal AIMP2 degradation, another lysosome inhibitor NH_4_Cl also blocked the effect of VPS35 on AIMP2 degradation (Supplementary Figures S1a and b). On the other hand, AIMP2 did not appear to affect VPS35 function as endogenous VPS35 levels were not altered by AIMP2 overexpression (Supplementary Figures S2a and b).

Lysosomal degradation pathways include macroautophagy and CMA.^26^ To determine which autophagic pathway is involved in AIMP2 degradation, SH-SY5Y cells were transfected with V5-VPS35 and AIMP2 stability was monitored when macroautophagy was blocked by 3-methyladenine (3-MA) treatment. Inhibition of macroautophagy had no effect on AIMP2 degradation and Lamp2a stabilization that were facilitated by VPS35 expression (Figures 1h and i). Taken together, these findings indicate that VPS35 is involved in the regulation of AIMP2 expression possibly through the CMA pathway.

### AIMP2 interacts with VPS35

**Figure 2:**
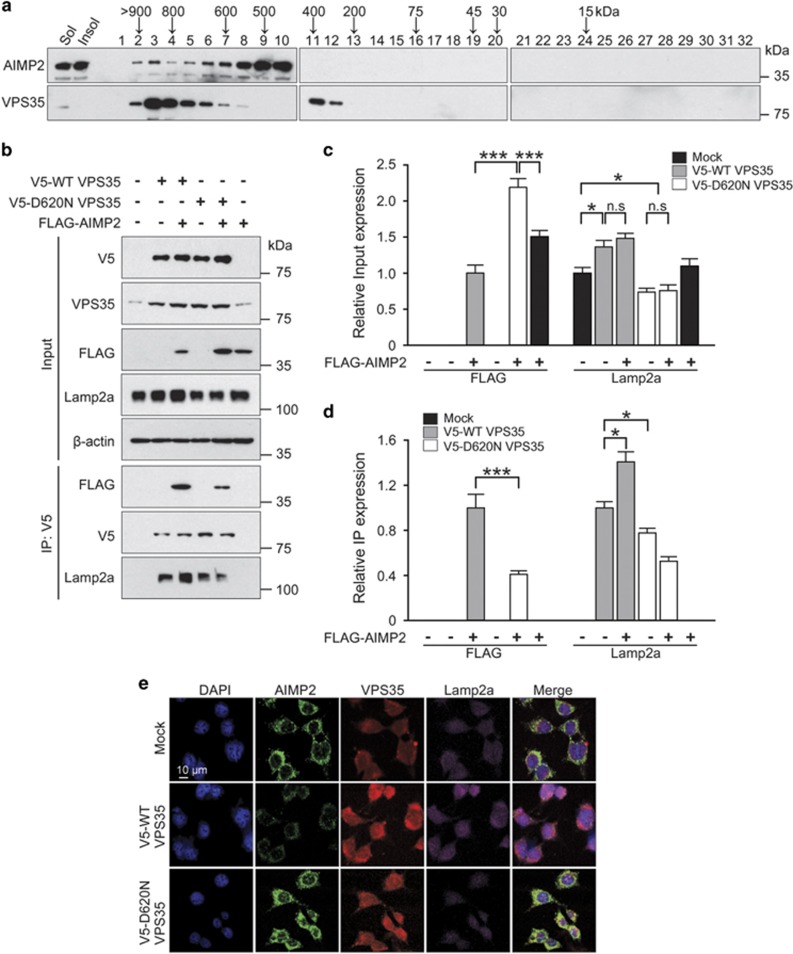
VPS35 is associated with AIMP2 and Lamp2a. (**a**) SH-SY5Y cell lysates were subjected to gel filteration chromatography. Fractions were analyzed by western blotting for the presence and location of AIMP2, and VPS35 using AIMP2, and VPS35-specific antibodies. The elution position of the molecular size marker is shown. There was co-elution in early fractions of AIMP2 with retromer complex protein, VPS35. (**b**) SH-SY5Y cells were transiently transfected with mock, V5-WT VPS35 or V5-D620N VPS35 with or without FLAG-AIMP2 for 48 h. Immunoprecipitate using anti-V5 antibody was analyzed by western blotting using antibodies against FLAG, V5 or Lamp2a. (**c**) Quantification and comparison of relative FLAG-AIMP2 and Lamp2a levels normalized to *β*-actin in input samples from experimental groups used in **b** (*n*=3). (**d**) Quantification of FLAG-AIMP2 and Lamp2a levels in anti-V5 immunoprecipitates from SH-SY5Y cells transiently transfected with the indicated constructs (*n*=3). (**e**) SH-SY5Y cells were transiently transfected with mock, V5-WT VPS35 or V5-D620N VPS35 for 48 h. Immunofluorescence microscopic images of AIMP2 (green), VPS35 (red) and Lamp2a (purple) in SH-SY5Y cells were obtained after staining with anti-AIMP2, anti-VPS35 and anti-Lamp2a antibodies. Each nucleus was stained with DAPI (blue). Each example shown is representative of three experiments. Scale bars: 10 *μ*m. ANOVA followed by *post-hoc* Bonferroni test was used for multiple group comparison. *P<0.05, ***P<0.001. n.s, not significant

VPS35 is the central largest subunit of retromer complex assembled with the other subunits including VPS26 and VPS29.^14, 27^ This trimer decides target protein recycling and degradation through target cargo binding.^14, 27^ To determine potential association of VPS35 and AIMP2, SH-SY5Y extracts were fractionated by gel filtration chromatography. Our results revealed that AIMP2, especially those in high molecular weight fractions, shared similar fractional location (fractions 2–8) with VPS35 (Figure 2a). We further characterized the interaction between VPS35 and AIMP2 by co-immunoprecipitation using SH-SY5Y cells transfected with FLAG-AIMP2 and V5-WT VPS35 or V5-D620N VPS35. Co-immunoprecipitation using antibody to V5 revealed that AIMP2 was strongly associated with V5-WT VPS35 (Figures 2b and d). Immunoprecipitation of V5-VPS35 also pulled down endogenous Lamp2a, a receptor for CMA degradation of aggregation-prone protein substrate. Consistent with previous report,^20^ WT VPS35 expression stabilized endogenous Lamp2a, whereas D620N VPS35 impaired Lamp2a recycling, leading to reduction of endogenous Lamp2a levels (Figures 2b and c). Mutant VPS35 expression was also correlated with its impaired association with AIMP2 and accumulation of AIMP2 when compared with WT VPS35 expression or mock DNA control transfection (Figures 2b and c). Finally, endogenously expressed VPS35 were colocalized with AIMP2 and Lamp2a in SH-SY5Y cells after co-labeling each protein (Figure 2e). Consistent with the immunoblot analysis (Figures 2b and c), WT VPS35 expression led to marked reduction of AIMP2 signals, whereas D620N mutant expression resulted in AIMP2 accumulation and even infiltration into the nucleus. Taken together, these results suggest that VPS35 may be associated with AIMP2 via Lamp2a and thereby regulating the stability of Lamp2a and the clearance of AIMP2 via lysosomal/autophagic pathway.

### VPS35 regulates AIMP2-induced cell death

**Figure 3:**
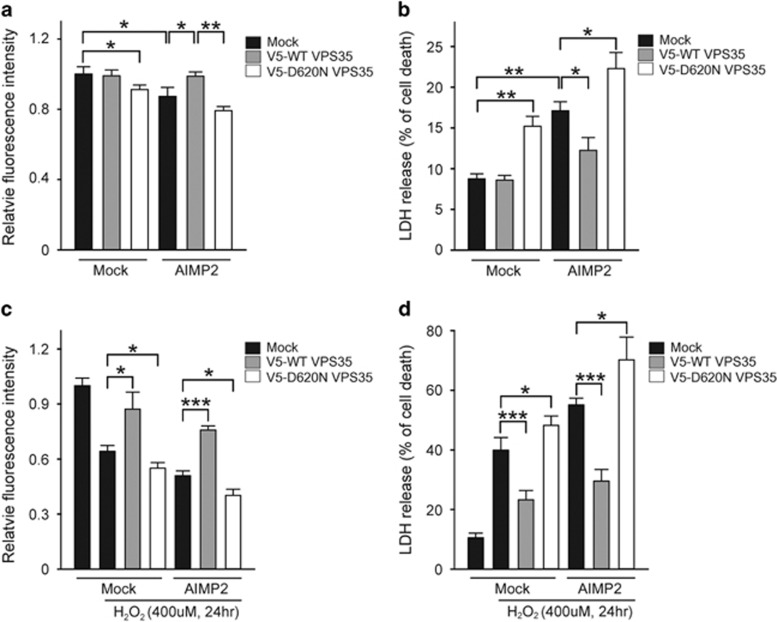
VPS35 attenuates AIMP2-induced cellular toxicity. (**a** and **c**) Cell viability based on alamarBlue assay. (**b** and **d**) Cell death based on LDH assay. SH-SY5Y cells were transiently transfected with WT VPS35 or D620N VPS35 with or without FLAG-AIMP2 for 48 h followed by cell viability assays (**a** and **b**). Transiently transfected SH-SY5Y cells were also challenged with 400 *μ*M H_2_O_2_ for 24 h followed by cell viability assays (**c** and **d**). The values are reported as mean±S.E.M. of three independent experiments with triplicate dishes. ANOVA followed by *post-hoc* Bonferroni test was used for multiple group comparison. *P<0.05, **P<0.01, ***P<0.001

Previously, we had shown that AIMP2 accumulation in SH-SY5Y cells or dopaminergic neurons *in vivo* is sufficient to cause cell death via PARP1 activation.^10^ As VPS35 overexpression reduced AIMP2 accumulation via lysosomal degradation, we hypothesized that overexpression of WT VPS35 could have protective effect against AIMP2-induced cell death, whereas D620N VPS35 may increase cell susceptibility to AIMP2 toxicity. To determine whether VPS35 can alter cell death in SH-SY5Y cells, we monitored cell viability by using two independent assays: alamarBlue assay and lactate dehydrogenase (LDH) release assay. Both assays demonstrated that cell death was increased when D620N VPS35 mutant was overexpressed (Figures 3a and b) where endogenous AIMP2 accumulation was also observed (Figures 1a and b). Consistent with our previous report,^10^ AIMP2 overexpression alone resulted in cell toxicity, which was markedly prevented by WT VPS35 overexpression. However, D620N VPS35 overexpression enhanced AIMP2-induced cell death (Figures 3a and b).

Oxidative stress is considered as a causative factor in PD pathogenesis. It has extensive interaction with PD-associated molecules, leading to cell death.^28, 29^ To further extend the potential therapeutic application of VPS35, we evaluated the ability of VPS35 expression in suppressing AIMP2 toxicity in the background of oxidative stress induced by hydrogen peroxide (H_2_O_2_). Similarly, cell death in response to H_2_O_2_ was monitored by using alamarBlue and LDH release assays in SH-SY5Y cells expressing mock or FLAG-AIMP2 together with WT or D620N VPS35. H_2_O_2_ treatment substantially increased cell death in both assays. AIMP2 expression further enhanced this toxicity (Figures 3c and d). Importantly, overexpression of WT VPS35 significantly ameliorated AIMP2 and/or H_2_O_2_-induced cell death, whereas overexpression of D620N VPS35 resulted in more cell death induced by AIMP2 and/or H_2_O_2_ (Figures 3c and d). These data together indicate that VPS35 expression protects SH-SY5Y cells from AIMP2- and oxidative stress-induced cell death, suggesting a potential therapeutic application of VPS35 in PD pathogenesis involving AIMP2 accumulation with or without oxidative stress.

### VPS35 regulates AIMP2 activation of PARP1

**Figure 4:**
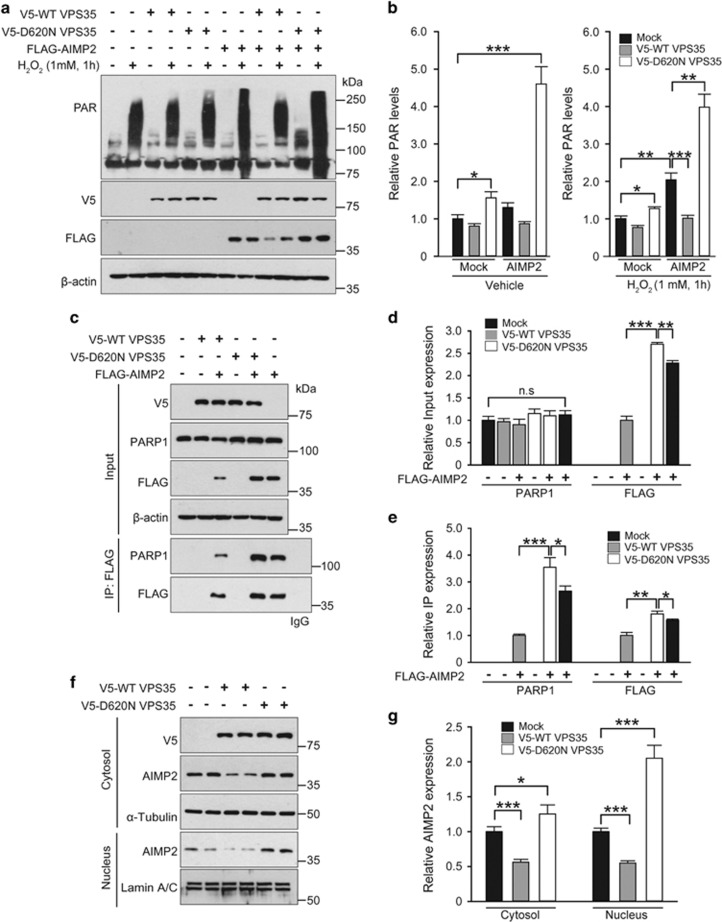
VPS35 suppresses AIMP2-potentiated PARP1 activation. (**a**) SH-SY5Y cells were transiently transfected with mock, WT VPS35 or D620N VPS35 with or without FLAG-AIMP2 for 48 h and treated either with 1 mM H_2_O_2_ for 1 h or with vehicle for 1 h as control. Total protein was extracted and immunoblotted with PAR, V5 and FLAG antibody to monitor their levels. *β*-Actin was used as an internal loading control. (**b**) Normalized levels of PAR in each experimental group (*n*=4 per group). In vehicle or H_2_O_2_-treated groups, relative PAR levels were compared with mock transfection control with vehicle or H_2_O_2_, respectively. (**c**) Anti-FLAG immunoprecipitation in SH-SY5Y cells transfected with indicated constructs was subjected to western blotting using antibodies against FLAG or PARP1. IgG was used as a negative control. (**d** and **e**) Quantification of relative PARP1 and FLAG-AIMP2 protein levels in input or anti-FLAG immunoprecipitates from SH-SY5Y cells transfected with the indicated combination of DNA constructs (*n*=4). (**f**) SH-SY5Y cells were transiently transfected with mock, V5-WT VPS35 or V5-D620N VPS35 for 48 h and harvested for subcellular fractionation. Distribution of V5-VPS35 and endogenous AIMP2 in cytosolic and nucleus fractions was determined by western blot. *α*-Tubulin was used as an internal loading control for the cytosolic fraction. Lamin A/C was used as an internal loading control for the nucleus fraction. (**g**) Normalized levels of endogenous AIMP2 in each subcellular fraction (*n*=4). Quantified data are expressed as mean±S.E.M. ANOVA followed by *post-hoc* Bonferroni test was used for multiple group comparison. *P<0.05, **P<0.01, ***P<0.001, n.s, not significant

AIMP2 accumulation can lead to dopaminergic cell death via nuclear translocation and subsequent binding to PARP1, resulting in PARP1 activation and PAR elevation, which mediate parthanatic cell death.^10^ To assess whether VPS35 can regulate PARP1 activation and PAR production by AIMP2, levels of PARsylated proteins were monitored by western blot using anti-PAR antibody. SH-SY5Y cells expressing FLAG-AIMP2 or mock as a control were subsequently challenged with H_2_O_2_ treatment. WT or D620N VPS35 was coexpressed to determine whether VPS35 could modulate PAR activation induced by AIMP2 and/or oxidative stress. PAR immunoblot analysis revealed that overexpression of D620N VPS35 mutant increased PARP1 activation compared with mock or WT VPS35 overexpression (Figures 4a and b). Elevation of PARsylated protein levels induced by FLAG-AIMP2 was reduced to basal levels by WT VPS35 coexpression. However, coexpression of D620N VPS35 further enhanced AIMP2-mediated activation of PARP1 (Figures 4a and b). VPS35 regulation of AIMP2-induced PARP1 activation was more evident in SH-SY5Y cells following H_2_O_2_ treatment that further increased the levels of PARsylated proteins. WT VSP35 mitigated AIMP2 enhancement of PARP1 activation in the background of H_2_O_2_ treatment compared with mock or D620N VPS35 mutant transfection with H_2_O_2_ treatment (Figures 4a and b).

PARP1 activation results from its interaction with AIMP2 in the nucleus.^10^ Therefore, we monitored subcellular distribution of AIMP2 and its interaction with PARP1 in response to VPS35 expression. Consistent with the notion that AIMP2 levels are reduced by VPS35, overexpression of WT VPS35 inhibited the binding of AIMP2 to PARP1 (Figures 4c and e) and reduced AIMP2 distribution to the nucleus fraction (Figures 4f and g). In contrast, overexpression of D620N VPS35 that resulted in more accumulation of AIMP2 enhanced the association of AIMP2 with PARP1 (Figures 4c and e) and increased AIMP2 expression in the nucleus fraction (Figures 4f and g). Taken together, these results suggest that VPS35 can modulate PARP1 activation by accelerating AIMP2 clearance, thus preventing AIMP2 from nuclear translocation or interaction with PARP1.

### Physiological role of VPS35 in AIMP2 clearance

**Figure 5:**
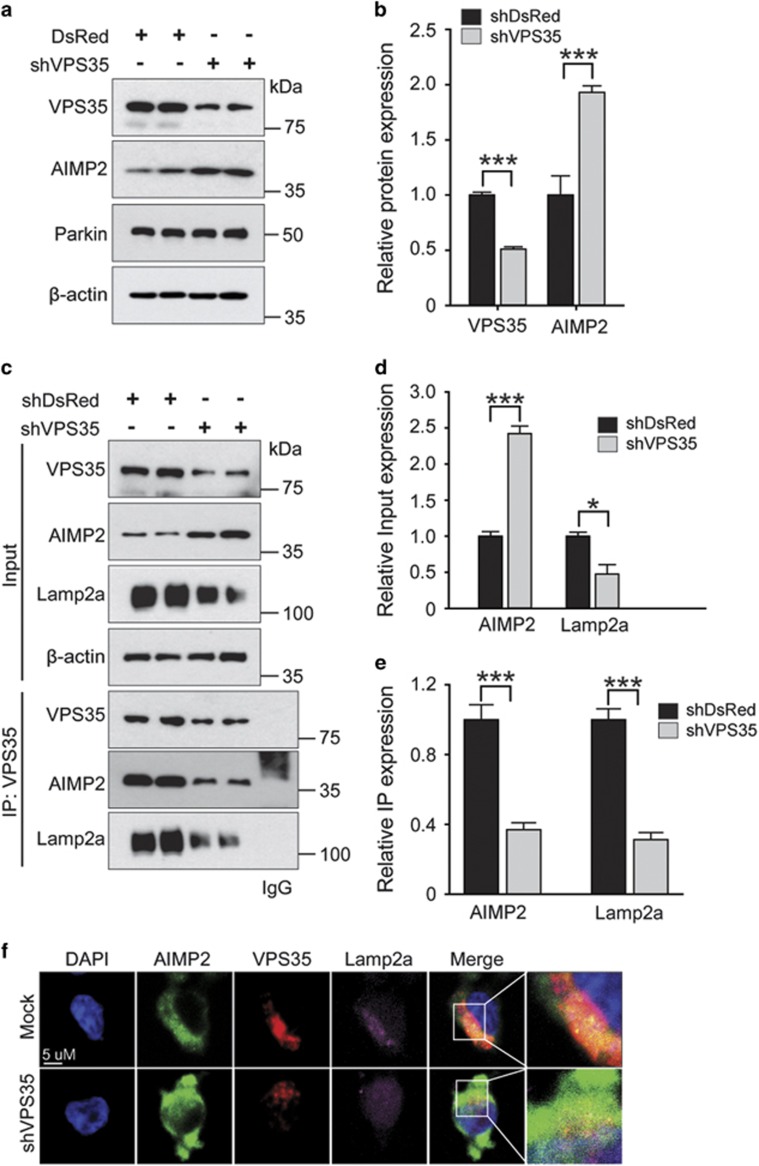
Loss of VPS35 leads to accumulation of AIMP2 and defective recycling of Lamp2a. (**a**) SH-SY5Y cells were transiently transfected with shRNA specific to VPS35 and DsRed (as a control) for 48 h. Total protein was extracted and immunoblotted with VPS35, AIMP2 and parkin antibodies to monitor their levels. *β*-Actin was used as an internal loading control. (**b**) Normalized levels of endogenous VPS35, AIMP2 and parkin in SH-SY5Y cells transfected with indicated constructs (*n*=4). (**c**) Immunoprecipitation of endogenous VPS35 in SH-SY5Y cells transfected with DsRed or shRNA specific to VPS35 was subjected to western blotting with antibodies against VPS35, AIMP2 and Lamp2a. IgG was used as a negative control. (**d** and **e**) Quantification of relative AIMP2 and Lamp2a protein levels in input or VPS35 immunoprecipitates from SH-SY5Y cells transfected with shDsRed control or shRNA to VPS35 (*n*=4). (**f**) Immunofluorescence microscopic images of AIMP2 (green), VPS35 (red) and Lamp2a (purple) in SH-SY5Y cells transfected with either shRNA control or shRNA to VPS35 for 48 h and subsequently stained with anti-VPS35, anti-AIMP2 and anti-Lamp2a antibodies. Each nucleus was stained with DAPI (blue). Each example shown is representative of three experiments. Scale bars: 10 *μ*m. Quantified data are expressed as mean±S.E.M. ANOVA followed by *post-hoc* Bonferroni test was used for multiple group comparison. *P<0.05, ***P<0.001 *versus* control

To determine whether endogenous VPS35 was involved in the process of AIMP2 turnover, we depleted VPS35 in SH-SY5Y cells by transiently transfecting shRNA to VPS35. VPS35 reduction by shRNA resulted in a significant increase of AIMP2 levels without affecting parkin expression (Figures 5a and b), supporting the role of endogenous VPS35 in the clearance of AIMP2. Moreover, ablation of VPS35 by shRNA reduced Lamp2a expression (Figures 5c and d). There was a reduction in association between AIMP2 and Lamp2a (Figures 5c and e). Supporting the notion that VPS35 is associated with AIMP2 under physiological condition, endogenously expressed VPS35 and AIMP2 colocalized in SH-SY5Y cells after co-labeling each protein (Figure 5f). shRNA-mediated downregulation of VPS35 resulted in diminished Lamp2a, enhanced AIMP2 signals and marked translocation of AIMP2 into the nucleus, indicating defective recycling of Lamp2a and inefficient clearance of AIMP2 (Figure 5f).

### AIMP2 accumulation downstream of VPS35 dysfunction has a role in cell toxicity

**Figure 6:**
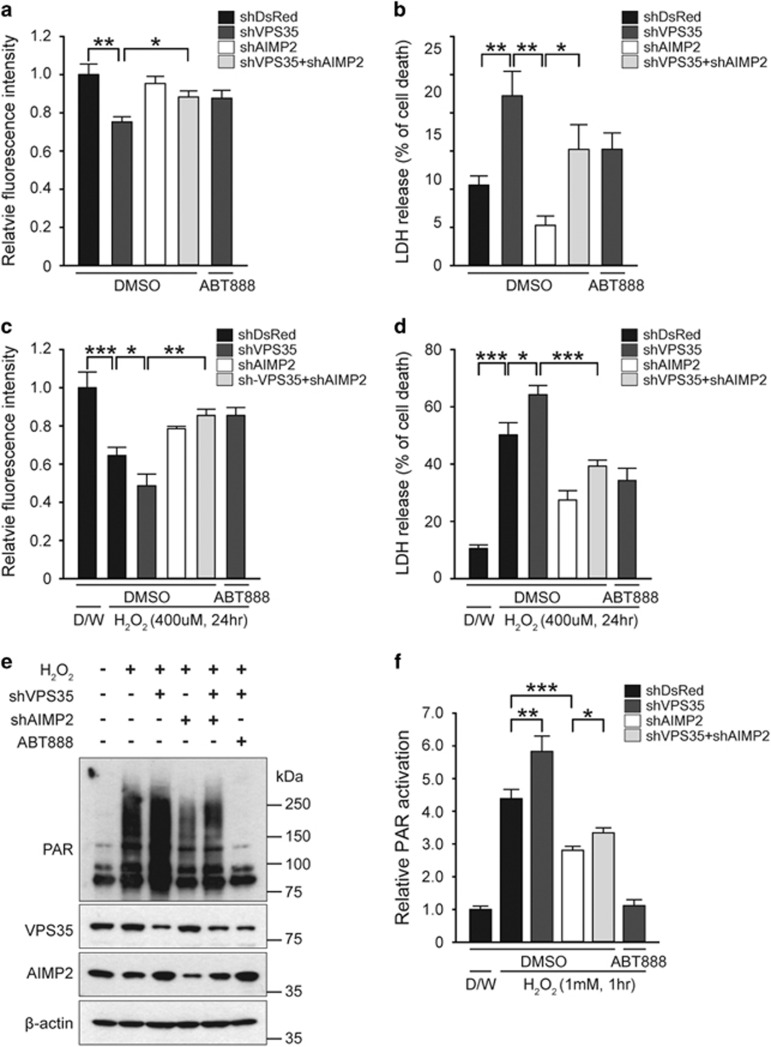
AIMP2 and PARP1 activation are required for cell toxicity downstream of VPS35 dysfunction. (**a** and **b**) SH-SY5Y cells were transiently transfected with shRNA to DsRed, VPS35 or AIMP2 for 48 h. Cell viability was determined by alamarBlue assay or LDH assay. PARP inhibitor (ABT888, 1 *μ*M) was treated to SH-SY5Y cells transfected with shRNA to VPS35 to assess the involvement of PARP1 activation in cell toxicity induced by VPS35 knockdown. (**c** and **d**) SH-SY5Y cells were transiently transfected with shRNA to DsRed, VPS35 or AIMP2 for 48 h followed by treatment with 400 *μ*M H_2_O_2_ for 24 h. Cell viability was determined by alamarBlue assay or LDH assay. PARP inhibitor (ABT888, 1 *μ*M) was treated to SH-SY5Y cells transfected with shRNA to VPS35 to assess the involvement of PARP1 activation in cell toxicity induced by VPS35 knockdown. D/W, distilled water. (**e**) Total protein was extracted from SH-SY5Y cells transfected with the indicated constructs followed by vehicle or 1 mM H_2_O_2_ treatment for 1 h. Protein levels were determined by western blot using PAR, VPS35 and AIMP2 antibodies. *β*-Actin was used as an internal loading control. (**f**) Normalized levels of PAR in each experimental group (*n*=4). The values are reported as mean±S.E.M. of three independent experiments with triplicate dishes. ANOVA followed by *post-hoc* Bonferroni test was used for multiple group comparison. *P<0.05, **P<0.01, ***P<0.001

As VPS35 functions in the clearance of AIMP2 under physiological condition, we sought to determine whether VPS35 dysfunction could affect PARP1 activation and cell death via AIMP2 accumulation. Supporting the protective role of VPS35 reported in previous studies,^22, 23, 24^ knockdown of VPS35 by shRNA resulted in an increase of cell death as assessed by alamarBlue and LDH release assays (Figures 6a and b). Cell death induced by VPS35 ablation was partially prevented by AIMP2 knockdown through shRNA (Figures 6a and b), indicating that AIMP2 mediates cell toxicity downstream of VPS35 deletion. The extent of cell protection rendered by AIMP downregulation was comparable to that of PARP inhibitor, ABT888 (Figures 6a and b). Moreover, enhancement of cell toxicity by H_2_O_2_ in the background of VPS35 knockdown was also attenuated by AIMP2 downregulation by shRNA or PARP inhibitor ABT888 (Figures 6c and d). PAR immunoblot analysis further revealed that downregulation of VPS35 increased H_2_O_2_-induced PAR activation that was largely blocked by AIMP2 shRNA or PAPR1 inhibitor (Figures 6e and f). Taken together, these results indicate that cell toxicity and PARP1 activation because of VPS35 deficiency is mediated by AIMP2 accumulation.

## Discussion

**Figure 7:**
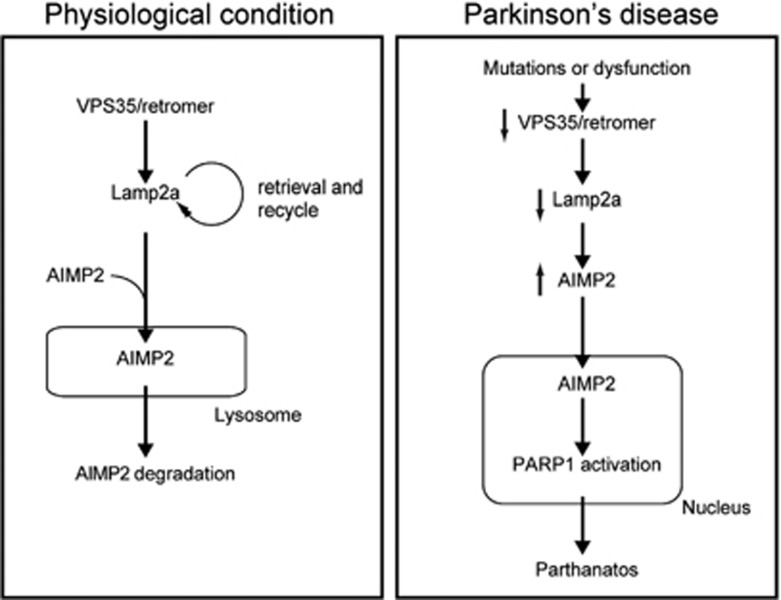
Schematic diagram illustrating AIMP2 clearance facilitated by VPS35-Lamp2a pathway. In PD condition where VPS35 function is compromised by its mutations or downregulation, Lamp2a recycling is impaired, leading to AIMP2 accumulation. High levels of AIMP2, in turn, lead to its nuclear translocation and thereby PARP1 activation causing PARP1-dependent cell death (parthanatos)

In this study, for the first time, we report that a retromer component VPS35 is associated with AIMP2 and that VPS35 regulates lysosomal degradation of AIMP2. Co-immunoprecipitation of VPS35 with AIMP2 and Lamp2a indicates that they are mutually associated with each other in the endosomal trafficking pathway. Based on previous report that Lamp2a stability is regulated by VPS35,^20^ it appears that VPS35/retromer can sort Lamp2a for recycling to the Golgi network. In lysosomes, Lamp2a tunnels lysosomal transport and degradation of misfolded proteins via CMA.^30, 31^ The identification of Lamp2a in association with AIMP2 suggests that misfolded AIMP2 could be degraded via the lysosomal pathway (Figure 7). Consistent with this notion and a previous report,^12^ lysosomal inhibitors blocked AIMP2 degradation facilitated by VPS35 overexpression. It is important to note that AIMP2 is found in Lewy body in association with *α*-synuclein in the brains of PD patient.^11, 12^ This indicates that AIMP2 is an aggregation-prone protein.^32^ In the brains of PD patients, parkin activity is compromised, leading to accumulation of its substrate proteins including AIMP2.^9, 11, 12, 33^ Under this condition, the activity of VPS35 could be critical to prevent abnormal accumulation of AIMP2 and its co-aggregation with *α*-synuclein.

AIMP2 stability was mainly assessed in a transient transfection setting because of the fact that prolonged and constitutive expression of mutant VPS35 in a stable cell line may lead to cellular toxicity. Nevertheless, further experiments using stable cell lines of VPS35 expression or knockdown would provide additional insight on VPS35-AIMP2 pathways by excluding potential variation in protein expression caused by transient transfection.

It is possible that the interaction between AIMP2 and VPS35 can affect the functions of VPS35/retromer. VPS35 dysfunction can lead to lysosomal dysfunction via abnormal targeting of various lysosomal proteases, thus contributing to AIMP2 accumulation in response to VPS35 knockdown. Interestingly, VPS35 knockout can lead to the downregulation of Lamp2a and the accumulation of *α*-synuclein.^20^ *α*-Synuclein elevation in turn inhibits CMA-mediated misfolded protein degradation, further exacerbating *α*-synuclein accumulation. This indicates that there is a reciprocal regulation and vicious cycle of *α*-synuclein/VPS35 pathology. Further studies are needed to determine the potential reciprocal regulation of VPS35 by AIMP2, although we did not observe any alterations in VPS35 expression when AIMP2 levels were increased.

AIMP2 expression can lead to PARP1 activation and dopaminergic neurodegeneration in mice.^10^ In this study, abnormal elevation of AIMP2 protein because of VPS35 deficiency resulted in its marked nuclear translocation and increased interaction with PARP1. This interaction stimulates PAR synthesis whose overproduction is responsible for parthanatic cell death.^10^ Given that AIMP2 levels are critical determinant of PARP1 activation and cell toxicity, modulating AIMP2 degradation pathway could be used as a potential strategy to prevent pathological consequences because of AIMP2 elevation (Figure 7). In this study, we showed that VPS35 overexpression accelerated AIMP2 degradation. Consistent with the role of AIMP2 in mediating cell toxicity, we showed that coexpression of WT VPS35 but not mutant VPS35 prevented cell toxicity caused by elevated AIMP2 in SH-SY5Y neuroblastoma cell line.

Downregulation of VPS35 resulted in the accumulation of AIMP2, suggesting that endogenous VPS35 was also involved in keeping optimal AIMP2 protein levels. It is likely that parkin and VPS35 function in separate degradation pathways to control endogenous AIMP2 expression whose dysregulation is pathologically detrimental in cell survival. Consistent with this notion, downregulation of VPS35 sensitized SH-SY5Y cells to parthanatic cell death caused by AIMP2 accumulation. It has been reported that VPS35 expression is decreased in the brains of AD and PD patients.^18, 19^ Indeed, expression of PD-linked mutant VPS35 (D620N) led to similar effects to VPS35 knockdown on AIMP2 accumulation and AIMP2-related cellular toxicity. Although it is controversial regarding the mode of action by D620N mutation in VPS35,^17, 22, 34^ our data support the notion that D620N VPS35 is a dominant-negative mutation especially on endosomal recycling of Lamp2a and regulation of AIMP2 stability. AIMP2 accumulation has been seen in degenerating ventral midbrains of postmortem PD brains.^9, 10, 11, 12^ Both parkin inactivation and VPS35 dysfunction could have contributed to the highly variable accumulation of AIMP2 in pathologically inflicted PD patients' brains. Considering well-accepted pathological role of protein aggregation and extensive association of VPS35 with other PD genes, VPS35 loss-of-function may be involved in the regulation of many pathological processes in PD. AIMP2 regulation by VPS35 could be an additional critical process especially relevant to dopaminergic cell death in PD. Therefore, preventing AIMP2-mediated PARP1 activation by modulating VPS35 function could be used as a potential therapeutic strategy for PD.

Given the fact that AIMP2 accumulation is found in both sporadic PD and genetic PD with parkin mutations, identification of alternative route of AIMP2 clearance is meaningful in that it provides additional pathway to prevent pathological AIMP2 accumulation. Pharmacological chaperone has been successfully developed to stabilize VPS35/retromer complex, thereby limiting amyloid precursor protein processing.^35^ As VPS35 reduction is also implicated in PD,^13, 21^ such stabilizer could prevent AIMP2 accumulation induced by VPS dysfunction and parkin inactivation. In addition to VPS35, parkin induction could prevent AIMP2 accumulation. Recently, it has been reported that diaminodiphenyl sulfone treatment can induce parkin expression via mild oxidative stress.^36^ It would be interesting to test these chemicals in animal models of AIMP2 accumulation to determine whether dopaminergic cell loss by AIMP2-activated parthanatos can be prevented in a synergistic manner.

## Materials and methods

### Antibodies

The following primary antibodies were used in this study: rabbit antibody to AIMP2 (1: 2000, ProteinTech Group, Rosemont, IL, USA), goat antibody to VPS35 (1:2000, Abcam, Cambridge, MA, USA), rabbit antibody to Lamin A/C (1:1000, Santa Cruz Biotechnology, Dallas, TX, USA), rabbit antibody to *α*-tubulin-1 (1:1000, Cell Signaling Technology, Danvers, MA, USA), mouse antibody to Lamp2a (1:1000, Abcam), mouse antibody to PAR (1:1000, Trevigen, Gaithersburg, MD, USA), mouse antibody to Parkin (Park8, 1:2000, Cell Signaling), mouse antibody to FLAG (M2, 1:5000, Sigma-Aldrich, St. Louis, MO, USA), mouse antibody to V5 (1:500, Invitrogen), horseradish peroxidase (HRP)-conjugated antibody to V5 (1:5000, Invitrogen), HRP-conjugated antibody to FLAG (1:5000, Invitrogen, Carlsbad, CA, USA), HRP-conjugated mouse antibody to *β*-actin (AC15, 1:20 000, Sigma-Aldrich). The following secondary antibodies were used: HRP-conjugated antibody to mouse IgG (1:5000, GE Healthcare, Pittsburgh, PA, USA), HRP-conjugated donkey antibody to rabbit IgG (1:5000, GE Healthcare), HRP-conjugated rabbit antibody to goat IgG (1:3000, Sigma-Aldrich), Alexa-488 conjugated antibody to rabbit IgG (H+L) (1:250, Thermo Fisher Scientific, Waltham, MA, USA), Alexa-568 conjugated antibody to goat IgG (H+L) (1:250, Thermo Fisher Scientific), Alexa-647-conjugated antibody to mouse IgG (H+L) (1:250, Thermo Fisher Scientific).

### Reagents

MG132, cycloheximide, NH_4_Cl, Pep A1, E64D, H_2_O_2_, ABT888 and 3-MA were purchased from Sigma-Aldrich.

### Plasmids

pcDNA-V5-*VPS35* was kindly provided by Dr. Matthew Farrer (University of British Columbia, Vancouver, BC, Canada). The following constructs used in this study were described previously pCMV-Tag2a- FLAG-*AIMP2*,^10^ and shRNA-dsRed.^6^ A plasmid containing *β*-galactosidase (empty vector) was used as a control in cell culture experiments. PLKO.1 shRNA plasmids encoding small interfering RNAs targeting AIMP2 and VPS35 were purchased from Genomics Resources in the Hit center (Johns Hopkins University, Baltimore, MD, USA). pLKO.1 shAIMP2 (TRCN0000072469) and pLKO.1 shVPS35 (TRCN0000099157) vectors were used to knockdown AIMP2 and VPS35 expression, respectively. As a control, shRNA-dsRed and short hairpin sequence (5′-AGTTCCAGTACGGCTCCAA-3′) were used.

### Site-directed mutagenesis

The expression plasmid V5-D620N *VPS35* mutant was generated using a QuikChange site-directed mutagenesis kit (Stratagene, La Jolla, CA, USA). Sequences were confirmed by automated DNA sequencing. Primers used were: F, 5′-GAAATCAGCAATTCCAAAGCA-3′ R, 5′-TGCTTTGGAATTGCGGATTTC-3′.

### Cell culture and transfection

Human neuroblastoma SH-SY5Y cells (ATCC, Manassas, VA, USA) were grown in DMEM containing 10% FBS (vol/vol) and antibiotics in a humidified 5% CO_2_/95% air atmosphere at 37 °C. For transient transfection, cells were transfected with indicated target vectors using Lipofectamine and Plus reagents (Invitrogen) according to the manufacturer's instructions. Unless otherwise indicated, lysates were prepared at 48 h after transfection.

### Real-time polymerase chain reaction (PCR)

Total RNA was extracted from cells with RNeasy Plus Mini Kit (Qiagen, Germantown, MD, USA). cDNA was synthesized from total RNA (1.5 *μ*g) using a First-strand cDNA synthesis kit (Invitrogen). Aliquots of cDNA were used as templates for real-time qRT-PCR. Relative quantities of mRNA expression levels were analyzed using real-time PCR (Applied Biosystems ABI PRISM 7700 Sequence Detection System, Applied Biosystems, Foster City, CA, USA). SYBR green ER reagent (Invitrogen) was used according to the manufacturer's instruction. The primer sequences for real-time PCR were: AIMP2-F, 5′-AGGTAAAGCCCTATCACGGG-3′ AIMP2-R, 5′-ACAGGTTAGACTCTTCCTGCAC-3′ GAPDH-F, 5′-TGTTTGTGATGGGTGTGAAC-3′ GAPDH-R, 5′-TACTTGGCAGGTTTCTCCAG-3′. Real-time PCR conditions were: denaturation at 94 °C for 5 min, 30 cycles at 94 °C for 45 s, annealing temperature (Tm) 60 °C for 30 s, and 72 °C for 30 s, followed by 5 min of extension at 72 °C. When the melting Tm was reached, double-stranded DNA was denatured and the SYBR was released, causing a marked decrease in fluorescence intensity. The rate of this change was determined by plotting the derivative of the fluorescence relative to the Tm (dF/dT) *versus* Tm using data analysis software provided by the real-time PCR instrument. The Tm at which a peak occurred on the plot corresponded to the Tm of the DNA duplex. GAPDH was used as an internal loading control for normalization.

### Gel filtration chromatography

Soluble protein extracts form SH-SY5Y cells were prepared in buffer A (50 mM Tris-HCl pH 7.5, 10% glycerol, 0.1 mM EDTA, 100 mM NaCl, 1 mM mercaptoethanol, 5 mM ATP plus a cocktail of inhibitors). Gel filtration chromatography was performed with a Superose-6 column by FPLC (GE Healthcare UK Ltd. Buckinghamshire, UK). The column was equilibrated with two column volumes of buffer A. One milliliter of each fraction (32 ml in total) was separated by SDS-PAGE on 12% gels. Fractionated proteins were detected with AIMP2, and VPS35 antibodies.

### Co-immunoprecipitation

For co-immunoprecipitation experiments, at 48 h post transfection, SH-SY5Y cells were washed with cold PBS and harvested in immunoprecipitation buffer (1% NP-40, 2 mg per ml aprotinin and 100 mg per ml PMSF in PBS). The lysate was then rotated at 4 °C for 1 h followed by centrifugation at 22 250 × *g* for 20 min. The supernatants were then combined with protein G Sepharose beads (Amersham Biosciences, Piscataway, NJ, USA) pre-incubated with antibodies against FLAG or V5 or VPS35 and mixed by rotating at 4 °C overnight. Protein G Sepharose was pelleted and washed twice with immunoprecipitation buffer followed by twice wash with 500 mM NaCl buffer. The precipitates were resolved on SDS-PAGE gels and subjected to immunoblot analysis. Immunoblot signals were visualized with chemiluminescence (Pierce, Rockford, IL, USA). Densitometric analyses of immunoreactive bands were performed using Image J software (NIH, Bethesda, MD, USA, http://rsb.info.nih.gov/ij/).

### Western blot analysis

Cells were harvested, washed twice with PBS and lysed with Pierce RIPA buffer (150 mM NaCl, 50 mM Tris, pH 8.0, 1% NP-40, 1% SDS and 0.5% sodium deoxycholate plus protease inhibitor mixture, Thermo Scientific) for 30 min on ice. Cell lysates were then prepared by centrifugation (22 250 × *g* at 4 °C for 20 min). Protein concentration was determined using Pierce BCA protein assay kit (Thermo Scientific). Equal amounts of protein (10–20 *μ*g) were resolved on 8–16 % SDS-PAGE and transferred to nitrocellulose membrane. After washing with TBST (Tris-buffer solution-Tween-20) (10 mM Tris-HCl (pH 7.6), 150 mM NaCl and 0.05% Tween-20), the membranes were blocked with 5 % skim milk for 1 h and incubated with an appropriate primary antibody at the dilution recommended by the supplier. The membrane was then washed and primary antibodies were detected with a HRP-conjugated secondary antibody. Immunoblot signals were visualized with chemiluminescence (Pierce). Densitometric analyses of the immunoreactive bands were performed using NIH Image J software. The ratios between each treated- and control samples were calculated for each individual experiment and expressed as a percentage compared with the control.

### Subcellular fractionation

Harvested cell pellet was mixed with buffer 1 (250 mM sucrose, 50 mM Tris-HCl, 5 mM MgCl_2_) in the presence of Halt protease inhibitor cocktail (Thermo Scientific) and incubated for 10 min on an end-over-end shaker followed by centrifugation at 1000 × *g* for 10 min. The supernatants containing cytosolic protein were transferred to cold tubes on ice. The pellet was suspended in buffer 2 (1 M sucrose, 50 mM Tris-HCl, 5 mM MgCl_2_) containing protease inhibitor cocktail and incubated on an end-over-end shaker for 30 min followed by centrifugation at 6000 × *g* for 10 min. The new supernatants containing membrane proteins were transferred to cold tubes on ice. The remaining pellet was suspended in buffer 3 (20 mM Tris-HCl, 0.4 M NaCl, 15 % glycerol, 1.5 % Triton X-100) containing protease inhibitor cocktail and incubated on an end-over-end shaker for 10 min. The supernatants containing nuclear proteins were collected after centrifugation at 6800 × *g* for 10 min.

### Pulse chase analysis

For pulse chase studies, SH-SY5Y cells were transiently transfected with plasmids as indicated. At 48 h after transfection, cells were treated with 100 *μ*g/ml cycloheximide, an inhibitor of protein biosynthesis. Cells were harvested at different time point as indicated. Cells were then lyses with RIPA lysis buffer (150 mM NaCl, 50 mM Tris, pH 8.0, 1% NP-40, 1% SDS, and 0.5 % sodium deoxycholate plus protease inhibitor mixture). Protein concentration of each sample was determined using BCA protein assay kit (Thermo Scientific). These samples were then subjected to western blot analysis. Densitometric analyses of immunoreactive bands were performed using NIH Image J software. Proteins levels were normalized to *β*-actin.

### Immunofluorescence

SH-SY5Y cells were plated onto poly-d-lysine-coated coverslips at 10 000 cells/cm.^2^ Cells were fixed with 4% paraformaldehyde in PBS and blocked in a solution containing 5% normal donkey serum (Jackson ImmunoResearch Laboratories, West Grove, PA, USA), 2% BSA (Sigma-Aldrich), and 0.1% Triton X-100 (Sigma-Aldrich) for 1 h at room Tm. Samples were then incubated with primary antibodies against AIMP2, VPS35 or Lamp2a at 4 °C overnight. Briefly, cells grown on coverslips were washed with PBS containing 0.1% Triton X-100 and incubated with anti-rabbit Alexa-488, anti-goat Alexa-568 and anti-mouse Alexa-647 antibodies (1:250, Thermo Scientific) at room Tm for 1 h. The coverslips were mounted with DAPI. Fluorescent images were obtained using a confocal microscope (Carl Zeiss, LSM 710, Thornwood, NY, USA).

### Cell viability assays

Cell viability was tested with two methods: alamarBlue assay (Invitrogen) and LDH assay (Sigma-Aldrich). Cell death was assessed through alamarBlue assay according to the manufacturer's protocol. LDH activity in culture medium representing relative cell viability and membrane integrity was measured using the LDH assay kit spectrophotometrically following the manufacturer's instructions.

### Statistics

Data are presented as mean±S.E.M. with at least three independent experiments. Representative morphological images were taken out of at least three experiments with parallel results. Unpaired two-tailed Student's *t*-test or analysis of variance (ANOVA) test followed by Bonferroni *post-hoc* analysis was used to assess statistical significance. Assessments with *P*-value <0.05 were considered as statistically significant.
