# Supplementary material for: VPS35 regulates parkin substrate AIMP2 toxicity by facilitating lysosomal clearance of AIMP2
Source: Cell Death Dis. 2017 Apr 6;8(4):e2741–. doi: 10.1038/cddis.2017.157 (PMC5477581; doi:10.1038/cddis.2017.157)
Supplement: Supplementary Information [file cddis2017157x1.pdf]

Supporting Information

VPS35 regulates parkin substrate AIMP2 toxicity by facilitating lysosomal clearance of AIMP2

Seung Pil Yun, Hyojung Kim, Sangwoo Ham, Seung-Hwan Kwon, Gum Hwa Lee, Joo-Ho Shin, Sang Hun Lee, Han Seok Ko, and Yunjong Lee

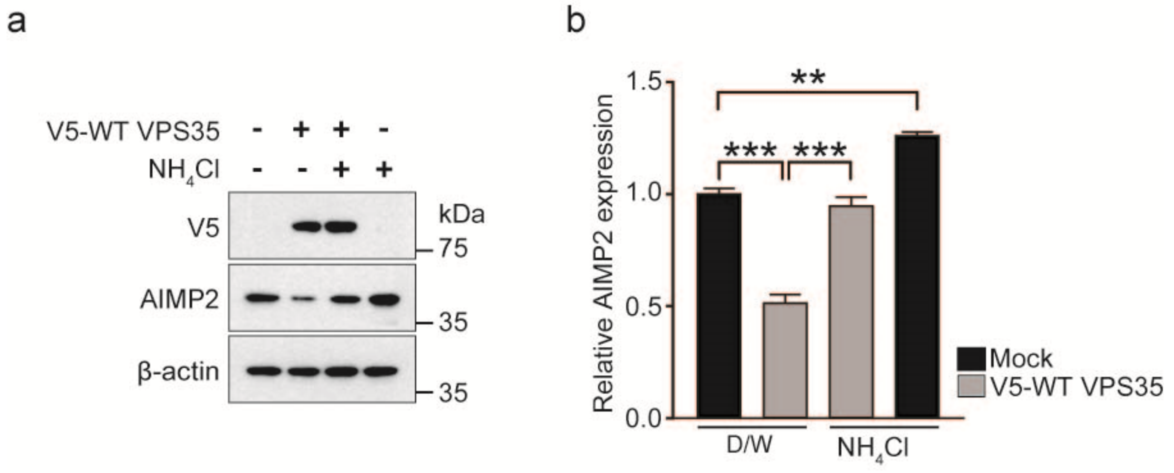

Figure S1. VPS35 enhances AIMP2 degradation via lysosome

(a) SH-SY5Y cells were transiently transfected with mock or V5-WT VPS35 and treated with NH_4Cl or distilled water as a vehicle (D/W). At 36 hrs post transfection, NH_4Cl (10 mM, 12 hrs) were used to treat cells. Total protein was extracted and immunoblotted with V5 and AIMP2 antibody to monitor their levels. β-actin was used as an internal loading control. (b) Normalized levels of AIMP2 expression in SH-SY5Y cells transfected with and treated with the indicated combination (n = 3). Error bars represent the mean ± S.E.M. Two-way ANOVA followed by Bonferroni post hoc analysis was used for multiple group comparison. $^{**}P < 0.01$, $^{***}P < 0.001$

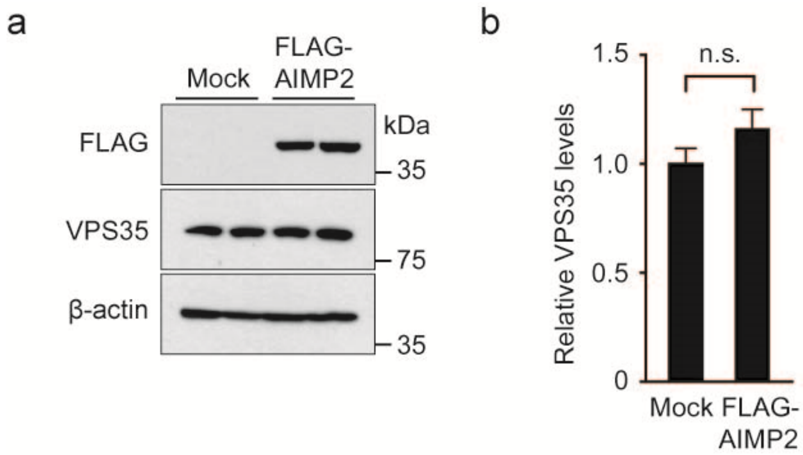

Figure S2. AIMP2 accumulation has no effect on VPS35 levels

(a) SH-SY5Y cells were transiently transfected with control or FLAG-AIMP2. At 48 hrs post transfection, total protein was extracted and immunoblotted with FLAG and VPS35 antibody to monitor their levels. β-actin was used as an internal loading control. (b) Normalized levels of VPS35 in SH-SY5Y cells transfected with mock or FLAG-AIMP2 constructs (n = 3). Error bars represent the mean ± S.E.M. Unpaired two-tailed Student's t test was used for statistical analysis.
